# TouchWGNN: spatio-temporal tactile perception for multimodal dexterous manipulation

**DOI:** 10.3389/frobt.2026.1791424

**Published:** 2026-06-26

**Authors:** Yu Ning, FuQiang Zhao, Qian Liu

**Affiliations:** Wireless Multimedia Technology Lab, School of Computer Science and Technology, Dalian University of Technology, Dalian, China

**Keywords:** dexterous manipulation, force and tactile sensing, graph neural networks, object pose estimation, reinforcement learning

## Abstract

Dexterous in-hand manipulation requires robotic hands to estimate object-state reliably under frequent occlusions, contact-rich interactions, and fast dynamics. Tactile sensing provides high-frequency, contact-specific feedback, although extracting useful representations from raw tactile signals and integrating them with vision and proprioception remains challenging. In this article, we present TouchWGNN, a multimodal dexterous manipulation framework that explicitly models tactile signals as a spatio-temporal graph. We first develop a low-cost distributed tactile sensor array for a five-fingered robotic hand, enabling real-time acquisition of normal forces from 113 sensing points distributed across key contact regions. We then construct a tactile graph in which taxels (or active contact points) are nodes with force features and 3D coordinates and edges encode spatial proximity and local force variation. A graph-based spatial encoder captures instantaneous contact geometry, and a temporal module models its evolution over time to refine object-state estimates. Finally, we fuse the tactile estimate with vision (point-cloud-based pose) and proprioception (joint states) for policy learning with reinforcement learning. Experiments on two in-hand manipulation tasks, cube reorientation and Baoding ball swapping, demonstrate that integrating raw tactile feedback with vision and proprioception improves manipulation performance compared with unimodal baselines.

## 1 Introduction

Endowing robots with human-level dexterity is a long-standing goal in robotics. Human hands combine a high number of degrees of freedom with strong multimodal perception, enabling robust manipulation under uncertainty (ElKoura and Singh, 2003; Yousef et al., 2011). In contrast, many robotic dexterous manipulation systems still rely primarily on vision. Vision provides global information, but it often requires multiple cameras and substantial computation; more importantly, it is sensitive to occlusions, lighting conditions, and latency, and it does not directly measure contact forces. These limitations motivate the use of tactile and proprioceptive signals to complement visual perception during contact-rich manipulation.

Tactile perception has therefore become a central topic in dexterous manipulation research. Recent work has begun to represent tactile readings with graph neural networks (GNNs), achieving promising results in tasks such as tactile-based object recognition (Gu et al., 2020) and tactile-driven in-hand control (Yang et al., 2023). However, most existing approaches process tactile data at a single time step or rely on static tactile snapshots. In dexterous manipulation, tactile signals evolve continuously as contacts appear, move, and disappear. Capturing this temporal evolution is critical for disambiguating object states and stabilizing control policies.

A second challenge lies in practical sensing. Tactile signals are spatially sparse yet temporally high frequency, which makes feature extraction non-trivial. In addition, many high-resolution tactile setups remain expensive or are limited to the fingertips, leaving large portions of the hand without coverage. Depending exclusively on tactile perception can also increase training cost and reduce efficiency because touch alone provides only local information (Kolamuri et al., 2021; Zhang et al., 2022). Inspired by this complementarity, we propose TouchWGNN, which refines vision-based pose estimates using spatio-temporal tactile graphs. We equip a five-fingered robotic hand with seven piezoresistive sensor units (113 taxels) covering the palm and fingers. Each time step yields a tactile graph whose nodes carry taxel forces and 3D locations, while edges capture local spatial relations. A geometry-aware graph network encodes instantaneous contact structure, and a temporal module aggregates history to stabilize pose estimates during contact-rich manipulation. We combine the tactile prediction with the visual estimate and the hand’s proprioception through a confidence-gated fusion module and learn manipulation policies with proximal policy optimization (PPO).

Contributions: The main contributions of this work are summarized as follows:

- A low-cost distributed tactile sensor array for a five-fingered hand (7 modules, 113 taxels) and a per-taxel calibration procedure for normal-force measurement.
- A dynamic tactile graph representation and an equivariant-attention spatial encoder (ET-Net) that predicts per-frame object pose from raw taxel forces and geometry.
- A spatio-temporal fusion network (STF-Net) that models tactile dynamics via event/trend temporal encoders and performs confidence-gated fusion with vision-based pose estimates.

## 2 Related work

### 2.1 Dexterous manipulation

Dexterous manipulation aims to enable multi-fingered robotic hands to perform complex in-hand operations such as reorientation, finger gaiting, and object stabilization. Early approaches relied on analytical models of contact mechanics and precise object models, which can be brittle under modeling errors and unmodeled contact uncertainties (Bhatt et al., 2022). More recently, learning-based approaches, particularly reinforcement learning and imitation learning, have become dominant, allowing policies to be learned directly from interaction data (Andrychowicz et al., 2020; Melnik et al., 2021; Yang et al., 2023). Despite this progress, matching human-level dexterity remains difficult, especially when perception is incomplete or unreliable. Most of these learning-based systems are primarily vision-centric or use only low-level contact indicators and thus remain vulnerable to visual occlusions, latency, and partial observability during contact-rich interactions. This motivates augmenting dexterous manipulation policies with richer tactile perception and explicitly modeling the spatio-temporal structure of contact signals.

### 2.2 Tactile sensing in robotic in-hand manipulation

Tactile sensing provides rich local measurements of contact forces and pressure distributions, which are essential for fine-grained control during manipulation. It has been used for object recognition, grasp stability prediction, and tactile-driven manipulation (Guzey et al., 2023; Yousef et al., 2011; Yang et al., 2023; Yin et al., 2023; Newbury et al., 2023). However, tactile data are often sparse across the hand surface and arrive at high frequency, making it challenging to extract spatially meaningful features and to integrate them over time. Recent deep tactile methods demonstrate that learned representations can significantly improve manipulation performance, but many assume dense, high-resolution, or multi-axis tactile skins and treat tactile arrays in isolation from other modalities. In contrast, our work focuses on a low-cost, normal-force-only distributed sensor array and explicitly combines tactile, vision, and proprioception within a unified spatio-temporal fusion framework.

### 2.3 Pose estimation

Accurate object pose estimation is critical for in-hand manipulation. Vision-based six degrees-of-freedom (6-DoF) pose estimation has advanced rapidly with deep learning and can be robust in cluttered scenes, but it remains sensitive to occlusions, lighting, and camera latency (Handa et al., 2020; Wen et al., 2024). Tactile sensing complements vision by providing reliable feedback during physical contact, motivating hybrid vision-tactile pose estimation pipelines. Foundation models for 6D pose, such as FoundationPose, provide strong generic visual estimates but do not exploit contact information, and purely tactile approaches often lack global geometric context. Our framework bridges this gap by using a vision-based pose estimator as a global prior and refining it with spatio-temporal tactile graphs and joint states.

### 2.4 Graph neural networks for in-hand manipulation

Graph representations naturally capture relational structure among interacting entities (Xie et al., 2019). GNNs have been widely adopted to model tactile arrays and contact patterns because nodes can represent taxels or contact points, and edges can encode spatial adjacency (Yang et al., 2023; Fan et al., 2022; Kulkarni et al., 2024). However, dexterous manipulation is inherently temporal: contacts evolve over time, and past interactions influence current decisions. This motivates models that jointly reason over spatial structure and temporal evolution. Existing graph-based tactile methods typically focus on static or short-horizon snapshots and often operate either in tactile-only or vision-only settings. In contrast, we construct spatio-temporal tactile graphs, process them with a geometry-aware spatial encoder and an event–trend temporal module, and fuse their predictions with vision and proprioception for policy learning in dexterous in-hand manipulation.

Across these four strands of prior work, a common picture emerges: dexterous in-hand manipulation requires robust state estimation under occlusions and contact-rich dynamics; vision-based pose estimators alone are vulnerable to such conditions; and tactile sensing is powerful but frequently under-utilized or assumed to rely on expensive multi-axis hardware. Our work addresses this gap by combining a low-cost, normal-force-only distributed tactile array with a spatio-temporal graph neural network and a vision-based pose prior, yielding a multimodal state estimator that is both hardware-efficient and well-suited to contact-rich dexterous tasks.

## 3 Methodology

### 3.1 Distributed tactile sensor array

#### 3.1.1 Configuration of the distributed tactile sensor array

**FIGURE 1:**
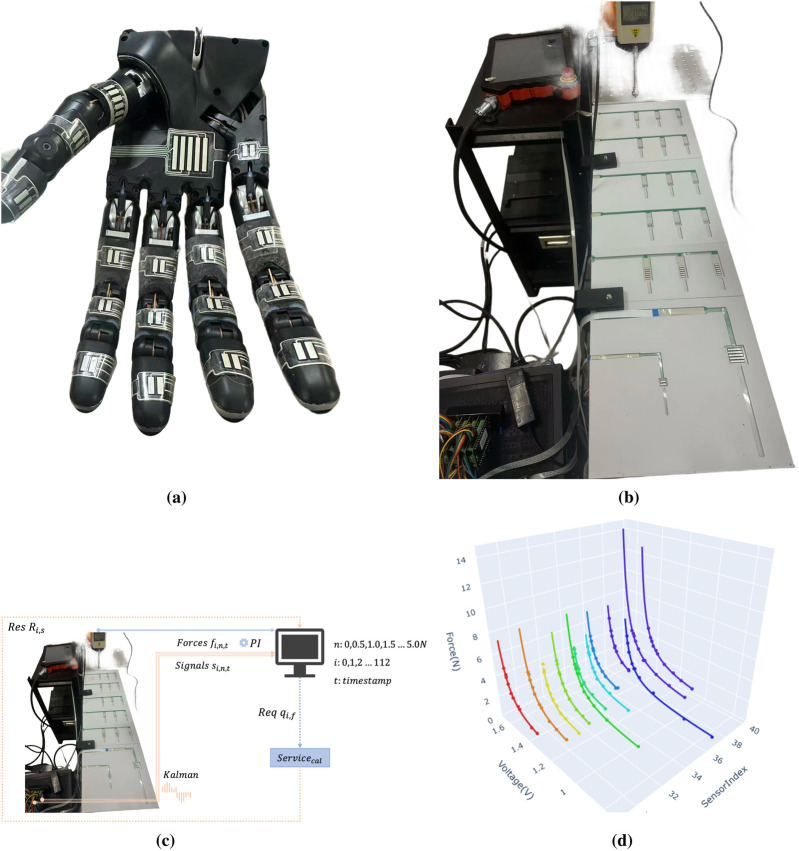
ShadowHand with sensors and calibration system. **(a)** ShadowHand with sensors, **(b)** calibration system, **(c)** calibration system data flow, and **(d)** sensor calibration result.

Seven tactile sensor array modules are distributed over the key contact areas of the hand, enabling real-time acquisition of normal-force information at these critical regions during manipulation. Specifically, a 6×6 array is mounted on the thumb, 2×6 arrays are mounted on the index, middle, ring, and pinky fingers, a 5×5 array is mounted on the main palm, and a 2×2 array is mounted on the sub-palm (the area below the pinky), as summarized in Table 1 and illustrated in Figure 1a. Specifically, the tactile sensors adopted in our system are piezoresistive sensors, whose mature fabrication process and simple working principle allow us to obtain a complete set of seven sensor array modules for only $7. Combined with data acquisition cards and simple post-processing filters, the system can be readily deployed in practical applications.

**TABLE 1:** Configuration of the distributed tactile sensor array.

[1.5 pt] **Finger**	Sensor array	Number of sensors
Thumb	6×6	36
Index finger	2×6	12
Middle finger	2×6	12
Ring finger	2×6	12
Pinky finger	2×6	12
Palm	5×5	25
Sub-palm	2×2	4
Total	7	113

We use four data acquisition cards to collect data from all sensors in parallel and transmit the data to the host computer through USB 3.0 interfaces. The collected data are then multiplied by the calibration matrix obtained in Section 3.1.2.5 to estimate the normal forces at each taxel.

#### 3.1.2 Calibration system

##### 3.1.2.1 Hardware setup

The proposed calibration system is illustrated in Figure 1b. It consists of four USB data acquisition (DAQ) modules, a custom 3D-printed calibration fixture, an HP-200 digital force gauge mounted on the end-effector of an AUBO-I10 robotic manipulator, a tactile sensor array, and a host computer responsible for data synchronization and logging. The calibration fixture is fabricated according to the geometric dimensions of the tactile sensors, ensuring that each sensor can be precisely and repeatably positioned in the fixture. A custom flange rigidly mounts the force gauge to the robot end-effector. A pad with the same size as a single taxel is attached to the tip of the force gauge probe so that each pressing operation applies force to exactly one taxel. During calibration, the robot approaches the sensor surface along the surface normal in the laboratory coordinate frame, ensuring that only normal force is applied to each taxel.

##### 3.1.2.2 Calibration point planning

Because the geometric structure of the sensor array and the structural parameters of the calibration board are known, the three-dimensional coordinates of each taxel in the experimental coordinate system can be computed from the spacing between adjacent taxels in the sensor array and their spatial locations on the calibration board. The inverse kinematics of the robot is then solved to obtain the joint configurations in which the end-effector reaches these coordinates, yielding the motion trajectory of the robot above each taxel. In total, 113 calibration positions $P=\left\{p_{1},p_{2},\ldots ,p_{113}\right\}$ are generated, each corresponding to one taxel in the tactile array.

##### 3.1.2.3 Robot force control

At each calibration position, the robotic arm performs a downward vertical motion along the z -axis. For each position, a set of discrete target normal forces $f^{*}\in F$ (e.g., {0,0.5,1,…,5} N ) is applied so that the calibration dataset covers a range of loads per taxel. The system employs a two-stage control strategy to achieve a stable and repeatable force-loading process. First, position control is used to gradually approach the sensor surface with the probe. When the force sensor detects a small contact force of 0.01 N, the controller switches to a proportional-integral (PI) force-control mode with gains $K_{P}$ , $K_{I}$ (see Table 2). Because including a differential term would make the controller excessively sensitive to sensor noise, differential control is not used. During the force-control phase, the contact force is regulated in closed loop using feedback from the force gauge, keeping the force applied to the sensor within ±0.01 N (i.e., within the tolerance $\Delta f_{\text{tol}}$ in Table 2) of the target value $f^{*}$ . To further enhance control stability, the following two strategies are employed:

- **Segmented control strategy**: use position control before contact and switch to force control after detecting a small contact force;
- **Integral clamping strategy**: limit the integral term in the PI controller to prevent integral saturation.

**TABLE 2:** Calibration parameters used in the tactile sensing experiments.

[1.5 pt] symbol	Value
Force tolerance $\Delta f_{\text{tol}}$	0.01 N
Temporal window t	1 s
Maximum duration $t_{\max}$	25 s
PI gains $K_{P}$	$6.0\times 10^{-5}\;m/N$
PI gains $K_{I}$	$3.0\times 10^{-5}\;m/\left(N\cdot s\right)$
1D Kalman filter Q	$10^{-3}$
1D Kalman filter R	$10^{-2}$
Sensor precision	0.005 N
Repeatability accuracy of the robotic arm	0.03 mm

During each pressing operation, the system continuously records the force readings from the force gauge and the tactile sensor signals.

##### 3.1.2.4 Data acquisition architecture

All tactile sensors are sampled in parallel using four DAQ modules connected to the host computer via a USB hub and a USB 3.0 interface. The force gauge sends its measurements to the host computer through a serial interface. To reduce time-synchronization errors between the force values and the sensor signals, all data timestamps are generated consistently when the host computer receives the data. The host computer runs two independent processes using the Robot Operating System (ROS) service communication mechanism.

**Force Acquisition Process.** The force acquisition process continuously receives force measurements from the force sensor. The host computer maintains a circular buffer with a temporal window of length t (Table 2) to cache the most recent force readings. If the measured force remains within ±0.01 N of the target value throughout this window, the force is considered stable. The average of the force measurements within the window is then taken as the recorded force for the current pressing operation. If this stability condition is not satisfied within a predefined maximum duration $t_{\max}$ , the robot maintains its current pose for an additional duration t , and the average force over this interval is used as the recorded force. Once the force value is determined, the force acquisition process acts as a client and calls the service provided by the tactile signal acquisition process. The client sends a request packet $\text{Req}\left(Q_{i,f}\right)$ , which contains the taxel index i and the corresponding force value f applied to that taxel. The server then returns a response packet $\text{Res}\left(R_{i,s}\right)$ containing the corresponding tactile sensor signal s . Finally, the host computer stores the data pair $\left(Q_{i,f},R_{i,s}\right)$ in the calibration dataset, where $Q_{i,f}$ encodes the index i of the taxel and the applied normal force f , and $R_{i,s}$ encodes the corresponding tactile sensor signal s .

**Tactile Signal Acquisition Process.** The tactile signal acquisition process continuously receives raw sensor signals from the DAQ modules. The host computer maintains a circular buffer with a temporal window of length t to store the most recent sensor measurements. Each incoming measurement is first processed using a 1D Kalman filter (process and observation noise parameters Q , R in Table 2) to reduce noise and obtain a more reliable estimate of the tactile signal. The filtered value is treated as the current signal measurement of the corresponding taxel. A dedicated acquisition thread is assigned to each DAQ module, and the buffer capacity for each thread is determined according to the number of taxels connected to that module. After collecting a predefined number of data frames, the thread writes the data into a shared buffer, marks the acquisition as complete, and pushes the data into the circular queue. The tactile signal acquisition process operates as a server, waiting for service requests from the force acquisition process. Once a request is received, the server retrieves the sensor data within the corresponding timestamp window from the circular queue and computes the average value of the signals. The averaged signal vector is then returned to the client as the response packet $\text{Res}\left(R_{i,s}\right)$ .

All parameters used in the calibration system are listed in Table 2. The complete procedure, including the loop over all 113 positions and multiple target force levels, is summarized in Algorithm 1.

Algorithm 1 **Automated tactile sensor calibration procedure. The algorithm iterates over 113 calibration positions and multiple target force levels, employing a two-stage control strategy that first approaches the sensor surface via position control and then switches to PI force control upon detecting a 0.01 N contact force. A stability criterion verifies that the applied force remains within ±0.01 N of the target over a time window before recording; otherwise, the robot holds its pose for an additional duration. For each stable pressing, the algorithm collects indexed force–signal calibration pairs through a service-based communication architecture and returns the complete calibration dataset D.**

**Input:** Calibration positions $P=\left\{p_{1},p_{2},\ldots ,p_{113}\right\}$ , target force set F (e.g., {0,0.5,1,…,5} N ), time window t , maximum stabilization time $t_{\max}$

**Output:** Calibration dataset D

1: Initialize empty dataset D←∅ .
2: **for** each calibration position $p_{i}\in P$ **do**
3:  **Step 1: Position and approach**
4:  Move the robot end-effector to position $p_{i}$ .
5:  Approach the sensor surface using position control.
6:  **for** each target force $f^{*}\in F$ **do**
7:   **Step 2: Force control and buffering**
8:   **if** contact force ≥0.01 N **then**
9:    Switch to PI force control.
10:   **end if**
11:   Regulate the contact force to maintain $f^{*}$ within ±0.01 N (integral clamping strategy).
12:   Continuously collect force measurements and store them in a circular buffer of length t .
13:   **if** force remains within $|f-f^{*}|\leq 0.01\;N$ throughout the window t **then**
14: f← average of force readings in the window t .
15:   **else**
16:    **if** stabilization time exceeds $t_{\max}$ **then**
17:     Hold the robot position for an additional duration t .
18: f← average of force readings in this interval.
19:    **end if**
20:   **end if**
21:   **Step 3: Tactile request and recording**
22:   Send service request $Req\left(Q_{i,f}\right)$ to the tactile signal acquisition process (taxel index i , force f ).
23:   Receive $Res\left(R_{i,s}\right)$ : s is the average of filtered tactile readings at index i over the same timestamp window t .
24:   Add the calibration sample $\left(Q_{i,f},R_{i,s}\right)$ to D .
25:  **end for**
26: **end for**
27: **return** D

##### 3.1.2.5 Calibration results

Based on the collected calibration pairs $\left(Q_{i,f},R_{i,s}\right)$ , we first analyze the relation between the applied force F and the measured voltage V of the force-sensitive resistors (FSRs) in the voltage divider circuit. According to Ohm’s law and the voltage divider principle, the output voltage is $V=V_{cc}\cdot \frac{R_{\mathrm{ref}}}{R_{\mathrm{fsr}}+R_{\mathrm{ref}}}$ . Because the FSR resistance $R_{\mathrm{fsr}}$ is approximately inversely proportional to the applied force $\left(R_{\mathrm{fsr}}\propto 1/F\right)$ , V increases with F and approaches the supply voltage $V_{cc}$ , leading to a pronounced asymptotic saturation behavior. To capture this nonlinear relationship, we fit the calibration data using the rational mapping in Equation 1.

$$F\left(V\right)=\frac{b\cdot V}{a-V}+c,$$ (1)

where a is the saturation voltage (close to $V_{cc}$ ), b is the gain coefficient controlling the sensitivity, and c is the zero-offset correcting the baseline. The fitting results are presented in Figure 1d, with a root mean square error (RMSE) of 0.0892 N and $R^{2}=0.9985$ . After calibration, we compute three values a , b , and c for each taxel and store them in a 113×3 calibration matrix. Then, once the 113 taxel signals are acquired, element-wise vectorized operations can be used to obtain the normal force applied to each of the 113 taxels.

### 3.2 Dynamic tactile graph builder

We introduce the dynamic tactile graph builder (DTGBuilder), which constructs a tactile graph at each time step from raw tactile readings. Inspired by PyTorch Geometric (Fey and Lenssen, 2019), we represent each graph as a data object with the attributes listed in Table 3. Node features store the current normal-force readings, and node positions store the 3D coordinates of taxels in the hand frame. We additionally include an episode-reset indicator to prevent temporal modules from mixing sequences across different simulation episodes.

**TABLE 3:** Input features and variables used by DTGBuilder.

[1.5 pt] variable	Symbol	Description
Node feature	x	Normal-force readings at the current time step
Node position	pos	3D coordinates of taxels
Reset event	$r_{\mathrm{event}}$	Binary indicator marking the end of an episode (for masking temporal models)

The node features are the normal forces applied to each taxel. Specifically, in the simulation environment, each taxel is modeled as a rigid body attached with small offsets to the outer surface of the ShadowHand links. We can conveniently obtain their global 3D coordinates and normal forces using the interfaces provided by the simulator. In the real-world setup, the coordinates of each taxel can be computed via forward kinematics based on the structural dimensions of the ShadowHand and its ROS-based interface that provides real-time joint angles, while the normal force for each taxel is obtained using the calibration results described in Section 3.1.2.5.

Although in this work, the node features contain only normal-force readings per taxel. This choice reflects the current hardware, which provides a single-axis calibrated output. Under the rigid-object conditions considered in our tasks, changes in the spatial pattern and temporal evolution of normal forces already provide informative cues about contact state transitions and object motion. Nevertheless, the proposed DTGBuilder is agnostic to the dimensionality of tactile features: future sensor upgrades that provide shear-force components or local torque can be incorporated by augmenting each node feature from a scalar normal force to a multi-dimensional tactile vector, without changing the graph construction or the downstream spatial–temporal encoders.

We construct two variants of graph data for different predictors. For the single-frame model, we keep only frames that contain at least one active taxel (i.e., non-zero contact force) because empty frames carry no instantaneous spatial information. For the sequence model, we keep every frame, including those without active taxels, because the absence of contact is itself a meaningful temporal signal (e.g., indicating transitions between contacts).

### 3.3 Spatial encoder

#### 3.3.1 Graph neural networks

**FIGURE 2:**
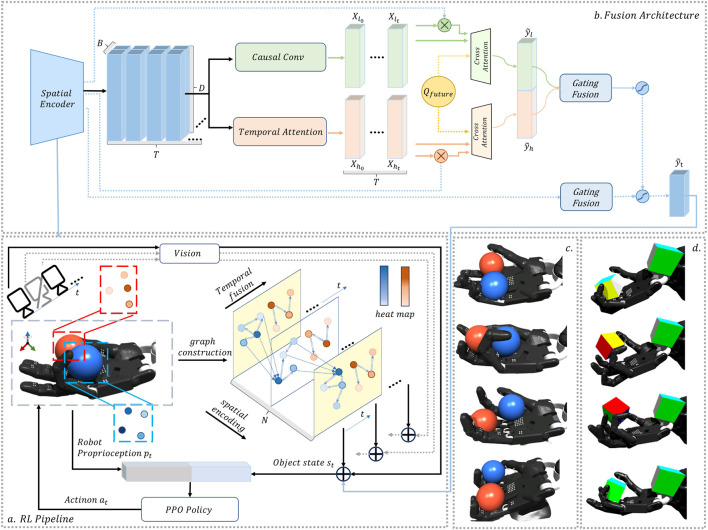
Architecture of the spatial–temporal fusion model.

Tactile information is distributed non-uniformly across the hand surface. We therefore adopt a graph neural network (GNN) to encode spatial relationships among taxels (Garcia-Garcia et al., 2019; Scarselli et al., 2009). Our spatial encoder, ET-Net, follows a hierarchical architecture built from an equivariant attention layer (EAL). Attention-based graph networks (e.g., GATv2 (Brody et al., 2021)) effectively model heterogeneous interactions, while equivariant coordinate updates (as in EGNN (Satorras et al., 2021)) introduce geometric inductive bias. EAL combines both: attention-based message passing is conditioned on geometry, and node coordinates are refined via equivariant updates, which are well-suited for tactile graphs where both force patterns and contact geometry matter. See Figure 2a for an illustration.

#### 3.3.2 Equivariant attention layer

Let the tactile graph at layer l be denoted as $G^{\left(l\right)}=\left(V^{\left(l\right)},E^{\left(l\right)}\right)$ , where each node $i\in V^{\left(l\right)}$ is associated with a feature vector $h_{i}^{\left(l\right)}\in R^{d}$ and a spatial coordinate $p_{i}^{\left(l\right)}\in R^{3}$ .

##### 3.3.2.1 Edge attributes

For each edge $\left(i,j\right)\in E^{\left(l\right)}$ , we compute the relative displacement using Equation 2:

$$r_{ij}=p_{i}^{\left(l\right)}-p_{j}^{\left(l\right)},\;d_{ij}=\|r_{ij}{\|}_{2},$$ (2)

and define a force-gradient term that characterizes local tactile variation using Equation 3:

$$g_{ij}=clip\;\left(\frac{\|h_{i}^{\left(l\right)}-h_{j}^{\left(l\right)}{\|}_{2}}{d_{ij}+\epsilon },\;0,\;S_{g}\right).$$ (3)

The final edge attribute is constructed using Equation 4:

$$e_{ij}=\left[r_{ij}\|g_{ij}\right].$$ (4)

##### 3.3.2.2 Attention-based message construction

For each node pair (i,j) , we construct an attention-based message that extends GATv2-style feature aggregation with geometry-conditioned edge attributes using Equation 5:

$$m_{ij}^{\left(l\right)}=\phi_{m}\left(h_{i}^{\left(l\right)},\;h_{j}^{\left(l\right)},\;e_{ij}\right),$$ (5)

where $\phi_{m}\left(\cdot \right)$ is implemented as a learnable multi-layer perceptron (MLP). This formulation can be viewed as a geometry-aware extension of GATv2 attention, in which edge attributes modulate the contribution of neighboring tactile nodes.

##### 3.3.2.3 Message aggregation

Messages from neighbors are aggregated as in Equation 6:

$$m_{i}^{\left(l\right)}=\underset{j\in N\left(i\right)}{\sum }m_{ij}^{\left(l\right)}.$$ (6)

##### 3.3.2.4 Feature update

Node features are updated via the residual rule in Equation 7:

$$h_{i}^{\left(l+1\right)}=h_{i}^{\left(l\right)}+\phi_{h}\left(h_{i}^{\left(l\right)},m_{i}^{\left(l\right)}\right),$$ (7)

where $\phi_{h}\left(\cdot \right)$ denotes a learnable update function.

##### 3.3.2.5 Equivariant coordinate update

Following EGNN-style coordinate refinement (Satorras et al., 2021), the spatial coordinates are updated using Equation 8:

$$p_{i}^{\left(l+1\right)}=p_{i}^{\left(l\right)}+\frac{1}{\left|N\left(i\right)\right|}\underset{j\in N\left(i\right)}{\sum }w_{ij}\;r_{ij},$$ (8)

where the displacement weight is computed using Equation 9:

$$w_{ij}=\sigma \;\left(\phi_{p}\left(h_{i}^{\left(l\right)},\;h_{j}^{\left(l\right)},\;e_{ij}\right)\right).$$ (9)

This formulation preserves translation equivariance and rotation equivariance of the coordinate updates. This geometric update is inspired by EGNN and injects equivariant geometric constraints into the attention-based message-passing process, rather than replacing the underlying GATv2 feature attention.

#### 3.3.3 Hierarchical pooling

Inspired by Yang et al. (2023), we adopt farthest point sampling (FPS) to progressively downsample the node set between layers, thereby capturing multi-scale structural information (Equation 10). To prevent excessive reduction, we enforce a minimum of n surviving nodes, which explicitly controls the downsampling strength.

$$V^{\left(l+1\right)}=\text{FPS}\left(V^{\left(l\right)},p_{i}^{\left(l\right)},\eta ,n\right)$$ (10)

where η is the downsampling ratio and n is the minimum number of surviving nodes. Finally, the global graph representation g is obtained via a global attention pooling layer, determined using Equation 11:

$$g=\underset{i\in V_{\mathrm{final}}}{\sum }\text{Softmax}\left({\text{MLP}}_{\mathrm{gate}}\left(h_{i}^{\left(l+1\right)}\right)\right)\cdot h_{i}^{\left(l+1\right)}.$$ (11)

#### 3.3.4 Object state prediction

Starting from the initial input N×P×C , where N is the number of graphs, P is the number of points, and C is the number of features dimensions, after pooling we obtain the global graph representation g:N×D , where D is the dimension of the global graph representation. We then employ an MLP to predict the object state using Equation 12, with nine dimensions per object: a 6-D rotation representation $\hat{r}$ and a 3-D position representation $\hat{p}$ ,

$$\left[\hat{p}\|\hat{r}\right]=\hat{o}=\text{MLP}\left(g\right).$$ (12)

Here, we treat the 3-D position representation as a displacement (i.e., velocity) with respect to the previous frame rather than as an absolute position. Because rotation is a non-Euclidean transformation, we adopt the continuous 6-D orthogonal representation (Zhou et al., 2018). Owing to the isotropic nature of a sphere, we cannot directly obtain its rotation information. However, its angular acceleration, which is induced by tangential forces, can be recovered from the 6-D rotation parameters; we therefore convert these parameters into angular acceleration (a second-order temporal difference) with the aid of PyTorch3D (Ravi et al., 2020). During both training and evaluation, we reconstruct absolute object poses by cumulatively integrating the predicted frame-to-frame displacements starting from the ground-truth pose at the beginning of each sequence and computing all position and angle errors with respect to these reconstructed absolute poses.

### 3.4 Spatial–temporal fusion architecture

#### 3.4.1 Spatial–temporal encoder

We propose STF-Net, a spatial–temporal encoder that captures both short-term, high-frequency tactile events and long-term, low-frequency trends. STF-Net uses (i) causal temporal convolutions to model local abrupt changes and (ii) masked temporal attention to model long-range dependencies. A fusion module then combines these cues with spatial predictions and vision-based pose estimates. See Figure 2b for an illustration.

##### 3.4.1.1 Temporal variations extraction

By analyzing the temporal evolution of an object’s state, we observe that state variations can be broadly categorized into two types of time-varying signals. **Events** correspond to high-frequency and abrupt changes, such as sudden translations or rotations caused by unexpected collisions. **Trends**, in contrast, represent low-frequency and gradual variations induced by deliberate interactions or persistent forces such as gravity.

**FIGURE 3:**
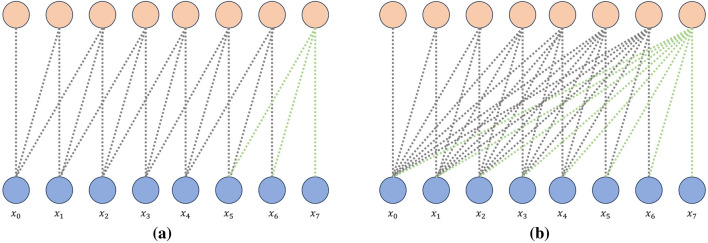
Causal convolution and temporal attention. **(a)** Causal convolution and **(b)** temporal attention.

We adopt different temporal mechanisms to model these two types of dynamics. We employ **causal convolution** with kernel size K and stride 1 to capture high-frequency events because its local receptive field restricts the amount of historical information involved at each time step, making it well-suited for modeling short-term, rapidly changing signals. For low-frequency trends, we utilize a **temporal attention mechanism**, which aggregates information over long temporal horizons and provides a global receptive field, as shown in Figure 3b, enabling the modeling of long-term dependencies and smooth temporal variations.

Causal convolution is a one-dimensional convolution with a strictly causal padding and indexing scheme, as shown in Figure 3a, Formally, given a temporal feature sequence $X=\left[x_{1},\ldots ,x_{T}\right]\in R^{T\times d}$ , where $x_{t}\in R^{d}$ denotes the feature vector at time step t , the causal convolution operating along the temporal dimension is defined using Equation 13:

$${\left(x*f\right)}_{t}=\overset{K-1}{\underset{k=0}{\sum }}f_{k}\cdot x_{t-k}.$$ (13)

The output of causal convolution, which is intended to capture event-level dynamics, is given by Equation 14:

$$X_{h}=\left[h_{1},\ldots ,h_{T}\right],\;h_{t}=\sigma \left({\left(X*f\right)}_{t}\right).$$ (14)

where σ is the activation function and $f=\left\{f_{0},\ldots ,f_{K-1}\right\}$ denotes a learnable causal convolution kernel.

For temporal attention, we compute the query, key, and value representations via learnable linear projections, as shown in Equation 15:

$$q_{t}=W_{Q}x_{t},\;k_{t}=W_{K}x_{t},\;v_{t}=W_{V}x_{t},$$ (15)

where $W_{Q},W_{K},W_{V}\in R^{d\times d}$ are learnable parameter matrices. In practice, causality and episode separation are jointly enforced by applying an attention mask M to the attention logits. Specifically, the mask is defined by Equation 16:

$$M_{t,i}=\left\{\begin{array}{cc} 0, & i\leq t\;\text{and both}\;t\;\text{and}\;i\;\text{belong to the same episode},\\ -\infty , & \text{otherwise}, \end{array}\right.$$ (16)

which prevents information leakage across future time steps and across different episodes. The output of causal temporal attention is given by Equation 17:

$$X_{l}=\left[{\tilde{x}}_{1},\ldots ,{\tilde{x}}_{T}\right],\;{\tilde{x}}_{t}=\overset{T}{\underset{i=1}{\sum }}\alpha_{t,i}v_{i},\;\alpha_{t,i}=\frac{\exp\left(q_{t}^{⊤}k_{i}+M_{t,i}\right)}{\sum_{j=1}^{T}\exp\left(q_{t}^{⊤}k_{j}+M_{t,j}\right)}.$$ (17)

Thus, we have extracted the event representation $X_{h}$ and trend representation $X_{l}$ from the original feature sequence.

##### 3.4.1.2 Spatial feature extraction and confidence estimation

For each frame, the DTGBuilder constructs a tactile graph $G_{t}=\left(V_{t},E_{t}\right)$ , where nodes represent taxel forces and edges encode spatial proximity. The spatial encoder predicts an object pose for each frame, producing a sequence of spatial pose predictions $\left\{{\hat{X}}_{t}^{S}\right\}$ . We further associate each prediction with a confidence score that measures its reliability. Concretely, the spatial encoder outputs both a mean pose ${\hat{X}}_{t}^{S}$ and a per-dimension log-variance $\log\sigma_{t}^{2}$ . From the log-variance, we derive an aleatoric confidence expressed by Equation 18:

$$c_{t}^{\text{alea}}=\sigma \left(-mean\left(\log\sigma_{t}^{2}\right)\right),$$ (18)

where σ(⋅) denotes the sigmoid function. In addition, we learn a student confidence head that maps the concatenation of the predicted pose and its log-variance to a scalar confidence (Equation 19),

$$c_{t}^{\text{stu}}=\sigma \left(f_{\text{stu}}\left(\left[{\hat{X}}_{t}^{S}\|\log\sigma_{t}^{2}\right]\right)\right),$$ (19)

where $f_{\text{stu}}\left(\cdot \right)$ is implemented as a lightweight MLP. During simulation training, ground-truth object poses $X_{t}^{*}$ are available, and we construct a teacher confidence (Equation 20):

$$e_{t}=\frac{1}{D}{\left\|{\hat{X}}_{t}^{S}-X_{t}^{*}\right\|}_{2}^{2},\;c_{t}^{\text{tea}}=\exp\left(-e_{t}\right)\;c_{t}^{\text{alea}},$$ (20)

that decreases monotonically with the squared error and is modulated by $c_{t}^{\text{alea}}$ . We then minimize a mean-squared error distillation loss expressed using Equation 21:

$$L_{\text{conf}}={\left\|c_{t}^{\text{stu}}-c_{t}^{\text{tea}}\right\|}_{2}^{2}$$ (21)

to encourage $c_{t}^{\text{stu}}$ to match $c_{t}^{\text{tea}}$ . At test time, including real-world deployment where ground truth is unavailable, we rely solely on the learned student confidence $c_{t}^{\text{stu}}$ , which depends only on the model outputs and their predicted uncertainty.

##### 3.4.1.3 Position embedding

We also incorporate position embedding for the total sequence length, including future time steps, to capture the temporal order of the features. The position embedding is a learnable parameter matrix $E\in R^{S_{\mathrm{total}}\times d}$ , where $E_{t}\in R^{d}$ denotes the position embedding at time step t . The position embedding is added to the feature vector at each time step using Equation 22:

$$x_{t}=x_{t}+E_{t},$$ (22)

where $x_{t}$ is the feature vector at time step t .

##### 3.4.1.4 Event–trend fusion, spatial–temporal fusion, and tactile–vision fusion

To predict current object states, we construct the query vectors by combining the temporal positional encoding of the target (current) frames with a learnable output query parameter. Specifically, the query at target (current) time step t is defined using Equation 23:

$$Q_{t}=LN\left(Q^{\text{out}}+{TE}_{t}^{\text{current}}\right),$$ (23)

where $Q^{\text{out}}$ is a learnable parameter and LN(⋅) denotes layer normalization.

Given the trend-level representation $X_{l}=\left[x_{1}^{l},\ldots ,x_{T}^{l}\right]$ and the event-level representation $X_{h}=\left[x_{1}^{h},\ldots ,x_{T}^{h}\right]$ , we apply two separate multi-head cross-attention modules via Equations 24, 25:

$${\hat{y}}_{l}={Attn}_{\text{trend}}\left(Q_{t},X_{l},X_{l}\right),$$ (24)

$${\hat{y}}_{h}={Attn}_{\text{event}}\left(Q_{t},X_{h},X_{h}\right),$$ (25)

where the same padding mask is used for both attention operations to ensure causality and episode isolation. The event-aware and trend-aware predictions are then fused using a learnable gating mechanism. We first concatenate the two representations: $z_{t}=\left[{\hat{y}}_{l}\|{\hat{y}}_{h}\right]$ , and compute a fusion gate via Equation 26 where σ(⋅) denotes the sigmoid function. The fused temporal prediction is given by Equation 27.

$$g_{t}=\sigma \left(W_{g}z_{t}+b_{g}\right),$$ (26)

$${\hat{y}}_{t}^{\text{ET}}=g_{t}⊙{\hat{y}}_{l}+\left(1-g_{t}\right)⊙{\hat{y}}_{h}.$$ (27)

##### 3.4.1.5 Unified gated fusion operator

We use a unified gated fusion operator to combine predictions from different sources. Given two candidate predictions a and b and an auxiliary confidence signal $c_{t}$ , the fusion is defined using Equations 28, 29:

$$\lambda_{t}=\sigma \left(W\left[a\|b\|c_{t}\right]+b\right),$$ (28)

$$Fuse\left(a,b,c_{t}\right)=\lambda_{t}⊙a+\left(1-\lambda_{t}\right)⊙b,$$ (29)

where σ(⋅) denotes the sigmoid function and [⋅‖⋅] denotes concatenation.

##### 3.4.1.6 Spatial–temporal fusion

Let ${\hat{y}}_{t}^{\text{S}}$ denote the spatial prediction and let ${\hat{y}}_{t}^{\text{ET}}$ the event–trend fused temporal prediction. The spatial–temporal fusion is computed using Equation 30:

$${\hat{y}}_{t}^{\text{ST}}=Fuse\left({\hat{y}}_{t}^{\text{S}},\;{\hat{y}}_{t}^{\text{ET}},\;c_{t}\right).$$ (30)

##### 3.4.1.7 Tactile–vision fusion

Let ${\hat{y}}_{t}^{\text{V}}$ denote the vision-based pose estimate and let ${\hat{y}}_{t}^{\text{ST}}$ denote the tactile spatial–temporal prediction. The final output is obtained using Equation 31:

$${\hat{y}}_{t}^{\text{TV}}=Fuse\left({\hat{y}}_{t}^{\text{ST}},\;{\hat{y}}_{t}^{\text{V}},\;c_{t}\right).$$ (31)

##### 3.4.1.8 Multi-supervision decoder

To effectively train the proposed spatial–temporal fusion architecture, we employ a multi-supervision decoding strategy that applies explicit supervision at multiple stages of the prediction pipeline. This design encourages each component to learn complementary representations and stabilizes optimization in long-horizon prediction.

###### 3.4.1.8.1 Spatial input supervision

The spatial encoder produces pose predictions for all frames in the sequence. Let ${\hat{X}}_{\text{in}}^{\text{S}}\in R^{E\times T_{\text{in}}\times D}$ denote the spatial predictions for the input frames and let $X_{\text{in}}^{*}\in R^{E\times T_{\text{in}}\times D}$ denote the corresponding ground-truth poses. Spatial predictions are supervised with the loss in Equation 32:

$$L_{\text{spatial}}={\left\|{\hat{X}}_{\text{in}}^{\text{S}}-X_{\text{in}}^{*}\right\|}_{2}^{2}.$$ (32)

###### 3.4.1.8.2 Confidence usage

During simulation-based training, teacher confidences constructed from ground-truth errors supervise the student confidence heads via distillation losses, so that the learned confidences correlate with actual prediction quality. During real-world inference, where ground truth is unavailable, only the learned student confidences, which are computed from the predictions and their log-variances, are used to gate temporal fusion and tactile–vision fusion.

###### 3.4.1.8.3 Temporal output supervision

The temporal encoder takes spatial features (or spatial predictions) from the input frames and produces target-frame pose predictions ${\hat{X}}_{\text{target}}^{\text{T}}\in R^{E\times T_{\text{target}}\times D}$ . We supervise them using the ground-truth target poses $X_{\text{target}}^{*}\in R^{E\times T_{\text{target}}\times D}$ (Equation 33):

$$L_{\text{temporal}}={\left\|{\hat{X}}_{\text{target}}^{\text{T}}-X_{\text{target}}^{*}\right\|}_{2}^{2}.$$ (33)

###### 3.4.1.8.4 Fusion output supervision

Let ${\hat{X}}_{\text{out}}\in R^{E\times T_{\text{out}}\times D}$ denote the final fused prediction and let $X_{\text{out}}^{*}\in R^{E\times T_{\text{out}}\times D}$ denote the corresponding ground-truth poses. The final fused output is supervised via Equation 34:

$$L_{\text{fusion}}={\left\|{\hat{X}}_{\text{out}}-X_{\text{out}}^{*}\right\|}_{2}^{2}.$$ (34)

###### 3.4.1.8.5 Overall objective

Because we initialize the visual branch with a pre-trained vision-based model, no additional supervision is applied to the visual pathway. The total training loss is a weighted combination of the above components (Equation 35):

$$L=\lambda_{\text{spatial}}L_{\text{spatial}}+\lambda_{\text{temporal}}L_{\text{temporal}}+\lambda_{\text{fusion}}L_{\text{fusion}}+\lambda_{\text{conf}}L_{\text{conf}}.$$ (35)

where $\lambda_{\text{spatial}}$ , $\lambda_{\text{temporal}}$ , $\lambda_{\text{fusion}}$ , and $\lambda_{\text{conf}}$ control the relative importance of each supervision signal. In all experiments, we apply stronger supervision on the spatial input and the final fused outputs while using moderate weights for the temporal prediction and confidence-distillation losses.

### 3.5 Reinforcement learning control policy

In this section, we will introduce our reinforcement learning and simulation setup.

#### 3.5.1 Problem formulation and environment setup

We formulate the problem as a Markov decision process (MDP) M=(S,A,P,R,γ) , where S is the state space, A is the action space, P is the transition probability, R is the reward function, and γ is the discount factor. The robot actor observe states (observations) $s_{t}$ every time step t and chooses an action $a_{t}$ computed by the policy network $\pi \left(a_{t}|s_{t}\right)$ to maximize the expected reward $r_{t}=R\left(s_{t},a_{t}\right)$ , discounted over the episode horizon T : $E\left[\sum_{t=0}^{T}\gamma^{t}r_{t}\right]$ . For both tasks, we use the same state space and action space.

- **State Space**: Consists of robot proprioceptive information $q_{t}\in R^{20}$ and object states ${\hat{X}}_{t}\in R^{N_{\mathrm{obj}}\times \left(3D\;position+6D\;rotation\right)}$ , where $N_{\mathrm{obj}}$ is the number of objects. The object states are supplied by the proposed spatial–temporal–vision fusion architecture.
- **Action Space**: $A=\left\{a_{t}\right\}$ , where $a_{t}$ is the joint angles of the hand.

##### 3.5.1.1 Cube reorientation task

The cube reorientation task is to rotate the cube to the target orientation, which is represented by the F2 distance between the current 6D and the target 6D rotation vectors being less than 0.1. Because we used a 6D rotation vector to represent rotations and orientations during the training phase to obtain continuous rotation representations, we use the F2 distance here to measure the similarity of rotations (Zhou et al., 2018). At the beginning of each episode, the cube is initialized at the palm center with 1% noise in its position and orientation. Figure 2d illustrates representative snapshots of the simulated cube reorientation process.

##### 3.5.1.2 Baoding-ball-swapping task

The Baoding ball swapping task involves swapping the positions of the two Baoding balls in the palm through continuous in-hand rotation. At the beginning of each episode, two Baoding balls are initialized on either side of the palm center with 1% noise in their positions.

Let $pos_{1,init}$ and $pos_{2,init}$ denote the initial positions of the two balls and define the initial relative position vector using Equation 36:

$$v_{\mathrm{init}}=pos_{1,init}-pos_{2,init}.$$ (36)

During manipulation, the current relative vector between two balls is expressed using Equation 37:

$$v_{\mathrm{cur}}=pos_{1,cur}-pos_{2,cur}.$$ (37)

We compute the rotation quaternion q that rotates $v_{\mathrm{init}}$ to $v_{\mathrm{cur}}$ , extract the rotation axis $n=\left(n_{x},n_{y},n_{z}\right)$ and angle θ , and represent this rotation as a rotation vector (Equation 38):

r=θn. (38)

To quantify the progress of the Baoding motion, we focus on the signed rotation around the z -axis,

$${\text{angle}}_{z}=r\cdot \left(0,0,1\right),$$ (39)

which measures how much the two-ball system has rotated within the palm.

**FIGURE 4:**
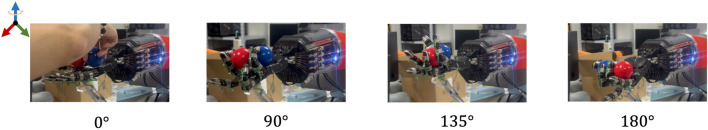
Baoding ball swapping task in reality.

A successful Baoding ball swap is declared when the current relative vector is approximately the reverse of the initial one, which is shown in Equation 40:

$$\|v_{\mathrm{cur}}+v_{\mathrm{init}}\|<\tau ,$$ (40)

where the τ value used in the experiments is 0.01, while both balls remain in contact with the hand. The hard task setting further requires that this swapped configuration be held for five consecutive time steps, as described in Section 3.5.2. Figure 2c illustrates representative snapshots of the simulated Baoding ball swapping process, and Figure 4 shows the corresponding behavior on the real system.

#### 3.5.2 Reward function

We design task-specific reward functions for the two tasks so that the robot policy learns to solve them as efficiently as possible.

In the cube reorientation task, the easy setting rewards reaching the target pose, whereas the hard setting additionally requires maintaining this pose for five consecutive time steps. Consequently, once the target orientation is reached, the policy must discover a stable configuration that keeps the cube in place.

$$\begin{array}{cc} R_{\mathrm{easy,t}}:=\; & w_{1}\cdot R_{\mathrm{dist,t}}+w_{2}\cdot R_{\mathrm{rot,t}}+w_{3}\cdot R_{\mathrm{action,t}}+w_{4}\cdot R_{\mathrm{timeout}}\\ & +w_{5}\cdot R_{rot\text{\_}error}+w_{6}\cdot R_{\mathrm{achieve}}+w_{7}\cdot R_{\mathrm{fall}}. \end{array}$$ (41)

Here, $R_{\mathrm{dist,t}}$ is the distance between the block and the hand and encourages the policy to approach and interact with the object. $R_{\mathrm{rot,t}}$ measures the change in the block orientation relative to its initial orientation and encourages exploratory rotation. $R_{\mathrm{action,t}}$ is a penalty on the control input that helps avoid unnecessary or overly aggressive actions. $R_{\mathrm{timeout}}$ is a terminal penalty for failing to complete the task by the end of the episode, which encourages early completion. $R_{rot\text{\_}error}$ measures the discrepancy between the current and target orientations and encourages the policy to rotate the block to the desired pose. $R_{\mathrm{achieve}}$ is a sparse bonus for successfully completing the task.

$$\begin{array}{cc} R_{hard,t}:=\; & w_{1}\cdot R_{\mathrm{dist,t}}+w_{2}\cdot R_{\mathrm{rot,t}}+w_{3}\cdot R_{\mathrm{action,t}}+w_{4}\cdot R_{\mathrm{timeout}}\\ & +w_{5}\cdot R_{rot\text{\_}error}+w_{8}\cdot R_{\mathrm{hold}}+w_{6}\cdot R_{\mathrm{achieve}}+w_{7}\cdot R_{\mathrm{fall}}. \end{array}$$ (42)

The reward for the hard setting is designed similarly to that of the easy setting, but it additionally rewards maintaining a stable pose. We introduce a holding-time bonus: let $HF_{\mathrm{now}}$ be the number of frames already held and let $HF_{\mathrm{desired}}$ be the required number of frames to remain stable. We define the hold-time ratio $HF_{\mathrm{ratio}}$ as in Equation 43 and compute the $R_{hold}$ term as in Equation 44:

$$HF_{\mathrm{ratio}}=\frac{HF_{\mathrm{now}}}{HF_{\mathrm{desired}}},$$ (43)

$$R_{hold}=HF_{\mathrm{ratio}}\times R_{achieve}\times HF_{\mathrm{desired}}.$$ (44)

Each additional stable frame increments $HF_{\mathrm{now}}$ ; when $HF_{\mathrm{ratio}}=1$ , the $R_{hold}$ term reaches its maximum.

The goal of the Baoding ball swapping task is to exchange the positions of the two balls as quickly as possible. The hard level additionally requires that the swapped configuration be held for five consecutive time steps; as in the block-orientation task, we use the same hold-time bonus for the hard level. Otherwise, both difficulty levels share the same reward function:

$$\begin{array}{cc} R_{t}= & w_{1}\cdot R_{\mathrm{spin,t}}+w_{2}\cdot R_{\mathrm{action,t}}+w_{3}\cdot R_{\mathrm{timeout}}+w_{4}\cdot R_{\mathrm{achieve}}\\ & +w_{5}\cdot R_{\mathrm{pos}}+w_{6}\cdot R_{\mathrm{contact}}+w_{7}\cdot R_{\mathrm{dist}}+w_{8}\cdot R_{\mathrm{fall}}, \end{array}$$ (45)

where $R_{\mathrm{spin,t}}$ is the reward for the actor to spin the two Baoding balls around the z -axis, encouraging the actor to perform the rotational motion. In practice, $R_{\mathrm{spin,t}}$ is implemented as a monotonically increasing shaping term of the signed rotation angle ${\text{angle}}_{z}$ defined in Equation 39 so that larger progress in in-hand rotation yields higher reward. $R_{\mathrm{action,t}}$ is the penalty for the robot action and helps avoid unnecessary actions. $R_{\mathrm{timeout}}$ is the penalty for not completing the task at the end of the episode. It encourages the actor to complete the task early. $R_{\mathrm{achieve}}$ is the reward for completing the task. It encourages the actor to complete the task. $R_{\mathrm{pos}}$ penalizes the actor when the midpoint between the two Baoding balls drifts too far from their initial midpoint, encouraging the actor to keep the balls centered. $R_{\mathrm{contact}}$ and $R_{\mathrm{dist}}$ encourage the actor to have increased contact with the balls and ensure that each contact only generates a reward for the nearby object. Its detailed computation is shown in Algorithm 2. Overall, the reward functions for the cube reorientation task in the easy and hard settings are summarized in Equations 41, 42, respectively, while the reward function for the Baoding ball swapping task is given in Equation 45. The coefficients used in the base reward functions are summarized in Tables 4, 5.

**TABLE 4:** Coefficients of the base reward function for the Baoding ball swapping task (shared by both easy and hard settings).

[1.5 pt] coefficient	$w_{1}$	$w_{2}$	$w_{3}$	$w_{4}$	$w_{5}$	$w_{6}$	$w_{7}$	$w_{8}$
Value	1.0	−0.0002	−1.0	250	−0.1	0.01	0.01	−1.0

**TABLE 5:** Coefficients of the base reward function for the cube reorientation task in both easy and hard settings.

[1.5 pt] coefficient	$w_{1}$	$w_{2}$	$w_{3}$	$w_{4}$	$w_{5}$	$w_{6}$	$w_{7}$	$w_{8}$
Value	5.0	1.0	−0.0002	−10	−1.0	500	−10	500

Algorithm 2 **Computation of tactile-weighted distance and contact rewards for the Baoding ball swapping task. For each object–sensor pair, the algorithm computes Euclidean distances and proximity scores, assigns each sensor to its nearest object, weights both distance and contact contributions using normalized taxel activations, aggregates per-object distance and contact rewards, and returns the task-level distance and contact rewards by averaging over all objects.**

**Input:** Object positions $O={\left\{x_{o}\right\}}_{o=1}^{O}$ , sensor positions $S={\left\{y_{s}\right\}}_{s=1}^{S}$ , tactile Sensing ${\left\{T_{s}\right\}}_{s=1}^{S}$

**Output:** Distance reward $R_{\mathrm{dist}}$ , Contact Reward $R_{\mathrm{contact}}$

1: **Step 1: Distance and Proximity Mapping**
2: **for** each object o∈{1,…,O} and sensor s∈{1,…,S} **do**
3: $d_{o,s}\leftarrow \|x_{o}-y_{s}{\|}_{2}$

4: $R_{\mathrm{prox,o,s}}\leftarrow \text{clip}\left(\frac{0.1}{4d_{o,s}+0.02},0,1\right)$

5:  **end** **for**
6: **Step 2: Nearest-Neighbor Association**
7: **for** each sensor s∈{1,…,S} **do**
8:  Find $o^{*}=\arg\min_{o}d_{o,s}$

9: $M_{s,o}\leftarrow 1$ if $o=o^{*}$ , else 0
10: **end** **for**
11: **Step 3: Weighting and Reward Aggregation**
12: **for** each object o∈{1,…,O} **do**
13: $T_{\mathrm{total,o}}\leftarrow \sum_{s=1}^{S}T_{s}\cdot M_{s,o}$

14:  **if** $T_{\mathrm{total,o}}>0$ **then**
15: $W_{s,o}\leftarrow \left(T_{s}\cdot M_{s,o}\right)/\left(T_{\mathrm{total,o}}+\epsilon \right)$

16:  **else**
17: $W_{s,o}\leftarrow 1/S$

18:  **end** **if**
19: $R_{o,dist}\leftarrow \sum_{s=1}^{S}R_{\mathrm{prox,o,s}}\cdot W_{s,o}$

20: $R_{o,contact}\leftarrow \text{clip}\left(\frac{1}{S}\sum_{s=1}^{S}T_{s}\cdot M_{s,o},0,1\right)$

21: **end** **for**
22: **return** $R_{\mathrm{dist}}=\frac{\alpha }{O}\sum R_{o,dist}$ , $R_{\mathrm{contact}}=\frac{\beta }{O}\sum R_{o,contact}$

#### 3.5.3 Domain randomization

To enhance policy robustness and sim-to-real transfer, we perform extensive domain randomization on observations, actions, gravity, hand tendon properties (damping, stiffness), hand degrees-of-freedom (DoF) properties (damping, stiffness), and all object attributes, including size, mass, friction, and initial pose.

#### 3.5.4 Training details

We train the actor-critic policy with proximal policy optimization (PPO). For the IsaacGym simulation, we set dt = 0.01667 s with two simulation substeps. control_frequencyInv = 3 corresponds to a 20 Hz control frequency in the real world.

## 4 Experiments

### 4.1 Experiment setup

We use the ShadowHand as the dexterous end-effector. The hand has five fingers and 20 actuated degrees of freedom, yielding a 20-dimensional action space. We integrate seven piezoresistive sensor units with a total of 113 taxels across the palm and fingers to measure normal forces at key contact regions, as shown in Figure 1a.

Simulation is implemented in Isaac Gym, where taxels are modeled to match the physical sensor layout. To reduce the sim-to-real gap, we develop a ROS-based calibration system, as shown in Figure 1b, using an AUBO-i10 manipulator and a force gauge. The manipulator presses each taxel while force readings are recorded alongside tactile outputs; we fit a per-taxel mapping and assemble a sensor-to-force calibration matrix. Once the 113 taxel signals are acquired, element-wise vectorized operations can be used to obtain the normal force applied to each of the 113 taxels. We also equip the end-effector with a wrist-mounted camera to perform visual inference for object pose estimation, which is then used for visual–tactile fusion. All experiments are repeated with three random seeds.

### 4.2 Reward function with different configuration

**FIGURE 5:**
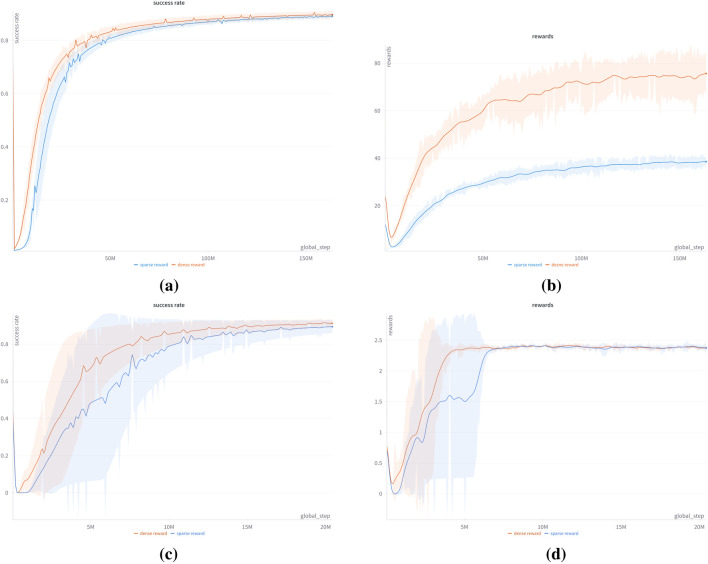
Performance of the policy on the cube reorientation task and Baoding ball swapping task with different reward configurations. **(a)** Cube reorientation task success rate, **(b)** cube reorientation task rewards, **(c)** Baoding ball swapping task success rate, and **(d)** Baoding ball swapping task rewards.

In this section, we compare sparse and dense reward designs for both tasks to validate the effectiveness of our shaped reward functions (Figures 5a–d).

For the cube reorientation task, the sparse configuration is obtained by zeroing out the shaping terms $R_{\mathrm{rot}}$ and $R_{\mathrm{fall}}$ in Equations 41 and 42, whereas the dense configuration keeps all terms active. As shown in Figure 5a, both variants eventually reach a high success rate, but dense rewards lead to consistently faster improvement and earlier saturation. The reward curves in Figure 5b further indicate that the dense configuration attains higher returns throughout training, demonstrating that rotation- and failure-related shaping accelerates policy learning, even though the final success rates of the two configurations are close.

For the Baoding ball swapping task, the sparse configuration is obtained by dropping the shaping terms $R_{\mathrm{pos}}$ , $R_{\mathrm{contact}}$ , and $R_{\mathrm{dist}}$ in Equation 45, while the dense configuration retains all components. Figure 5c shows that dense rewards again yield faster growth in success rate and earlier convergence than the sparse counterpart. Although the final average rewards in Figure 5d are similar, the dense configuration reaches its plateau significantly sooner, indicating more sample-efficient learning under the shaped reward.

### 4.3 Policy performance under different mask rates

**FIGURE 6:**
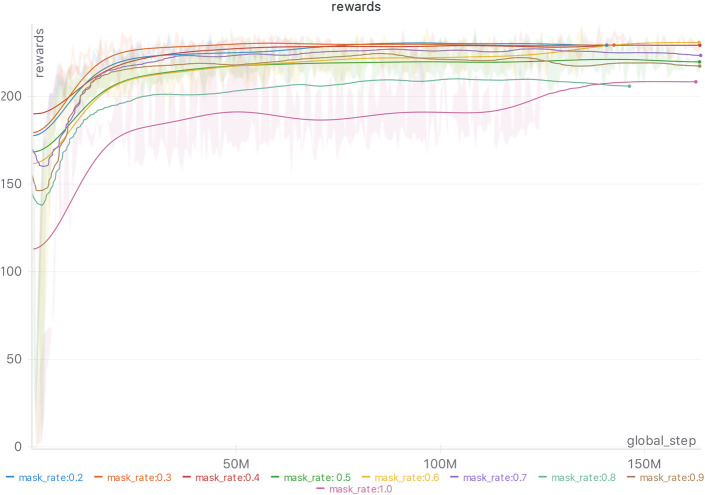
Observation masking.

To simulate the low-frequency sampling and latency inherent in real-world visual perception, we evaluate the policy’s robustness by randomly masking object-state observations. Specifically, at each timestep t , the current pose estimate is retained with probability p , and otherwise replaced with the “stale” observation from t−1 . In this setup, p=0 represents the most extreme case of latent pose observations. We conducted ablation experiments across a range of probabilities p∈{0,0.1,…,0.8} , keeping all other experimental configurations identical. As illustrated in Figure 6, the policy performance exhibits a clear upward trend as the masking rate decreases (i.e., as p increases). The significant degradation in performance as p approaches 0 underscores that frequent and accurate updates of the object state are indispensable for achieving high-performance dexterous manipulation.

### 4.4 Learning control policy with a different perception model

In this subsection, we evaluate the performance of various perception models across different tasks within the reinforcement learning (RL) framework. During the experiment, we collect object-state and tactile readings at every simulation step; every 100 steps, we train the spatial encoder alone for three epochs and then validate once, using its state prediction as the observation fed to the policy network, which then generates the appropriate control actions for the robot hand. Figure 2a shows the RL pipeline.

**FIGURE 7:**
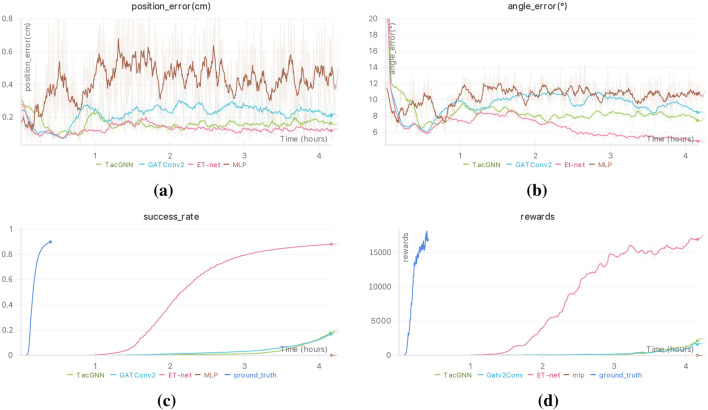
Simple Baoding swapping task. **(a)** Position error, **(b)** angle error, **(c)** success rate, and **(d)** rewards.

We first compare different spatial encoders on the simple Baoding ball swapping task. The resulting control performance is summarized in Figure 7. As shown in Figures 7a,b, our ET-Net method achieves the lowest position and angle prediction errors among all spatial encoders. Figures 7c,d show that ET-Net also achieves the fastest convergence in success rate and the highest cumulative rewards, yielding behavior closest to the unmasked oracle setting. To save computation, we stop training once the success rate reaches 0.9, which makes some learning curves in Figures 7c,d appear truncated, but this early stopping does not affect the final performance of any methods.

**FIGURE 8:**
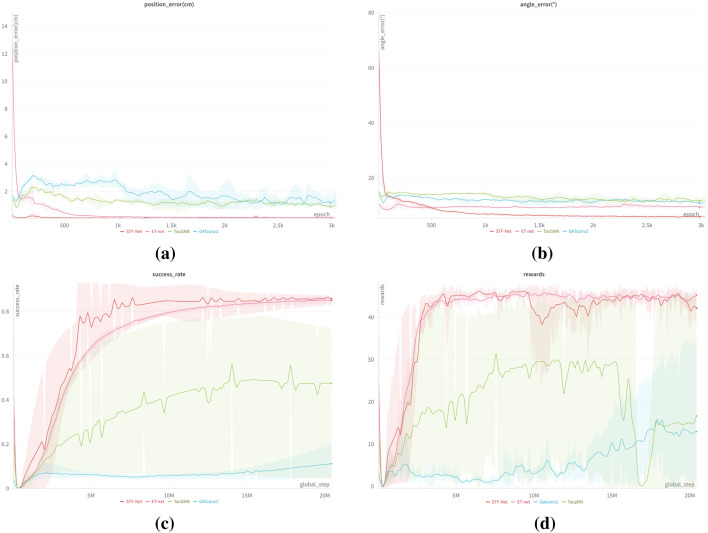
Simple cube reorientation task. **(a)** Position error, **(b)** angle error, **(c)** success rate, and **(d)** rewards.

We then evaluate STF-Net on the cube reorientation task. Figures 8a,b show that, compared with spatial-only predictors, the spatio-temporal fusion model further reduces both position and angle errors. In Figures 8c,d, STF-Net also achieves faster convergence in success rate and higher final rewards. Because the hard cube reorientation setting is intrinsically more challenging than the simple setting, most purely spatial baselines exhibit limited performance, whereas STF-Net still maintains a clear advantage in learning speed; although its reward curve in Figure 8d shows slight fluctuations, its overall trend remains higher than that of the spatial-only baselines.

Finally, Table 6 summarizes the final position error, angle error, success rate, and average episode length across both the cube reorientation and the Baoding swapping tasks and both difficulty levels. As shown in the table, our method STF-Net achieves the best position error, angle error, and average episode length across all tasks and difficulty levels.

**TABLE 6:** Performance of different perception models across tasks and difficulty levels.

Task	Level	Model	Position error (cm) ↓	Angle error °↓	Success rate ↑	Average steps ↓
Cube reorientation	Simple	GATConv2	1.166	11.63	84.33	336.87
		TacGNN	0.885	10.90	85.64	330.59
		ET-Net	0.096	9.39	87.81	250.44
		STF-Net	**0.071**	**5.93**	**90.56**	**244.20**
Cube reorientation	Hard	GATConv2	1.204	11.39	80.06	426.29
		TacGNN	1.030	12.56	79.17	424.90
		ET-Net	0.334	9.87	85.28	399.29
		STF-Net	**0.186**	**8.26**	**88.11**	**387.66**
Baoding swapping	Simple	GATConv2	0.148	12.01	94.01	45.83
		TacGNN	0.103	14.80	93.12	47.59
		ET-Net	0.074	10.85	96.37	29.28
		STF-Net	**0.042**	**8.71**	**98.24**	**26.55**
Baoding swapping	Hard	GATConv2	0.088	19.51	88.12	96.79
		TacGNN	0.144	20.42	88.23	95.28
		ET-Net	0.085	14.71	91.25	85.33
		STF-Net	**0.041**	**12.89**	**94.71**	**80.61**

However, we find that as the task difficulty increases, the performance of all methods declines. Notably, in the hard Baoding swapping task, STF-Net achieves lower position error than other methods and itself in the simple task. However, its success rate still decreases, indicating that the temporal fusion model cannot completely overcome the influence of task difficulty on the final performance. As the primary difference between the hard and simple tasks is whether to maintain the target position after achieving the goal, this further demonstrates two points:

- **Advantage of spatio-temporal fusion**: The spatial–temporal fusion architecture can capture the temporal information of the object movement, which is more sensitive to the object movement speed, achieving better performance.
- **Influence of task difficulty**: Although the spatial–temporal fusion architecture can achieve better performance, it cannot completely overcome the influence of task difficulty on the final performance. The success rate continues to decrease, indicating that the task difficulty remains the primary factor affecting the final policy performance of the dexterous manipulation task.

In addition, we observe that for the cube reorientation task, both ET-Net and STF-Net exhibit a more pronounced performance advantage over the other two baselines than in the Baoding ball swapping task, regardless of the difficulty level. We attribute this disparity to the inherent nature of the tasks: the cube reorientation task involves significantly more diverse and complex object trajectories. Because ET-Net and STF-Net possess superior capabilities in modeling dynamic motion and temporal dependencies, they are better equipped to capture the intricate state transitions of the cube. This demonstrates that our spatio-temporal modeling approach is well-suited for tasks with diverse trajectories and rapidly changing contact patterns.

### 4.5 Real robot experiment

Finally, we evaluate the performance of our proposed method on a physical robot platform. Our policy was successfully transferred to the real-world robot system as a result of leveraging extensive domain randomization during the training phase. A successful trial in the real robot is shown in Figure 4, in which the robot shows a similar performance to the simulation. This verifies that the learned tactile feature and policy can be applied to a real robot.

## 5 Conclusion

We presented TouchWGNN, a multimodal framework that uses spatio-temporal tactile graphs to refine object pose estimates and to improve dexterous in-hand manipulation. A distributed tactile sensor array provides high-frequency contact feedback across the hand surface. ET-Net encodes instantaneous contact geometry, and STF-Net models temporal evolution and fuses tactile predictions with vision and proprioception via confidence-gated fusion. Experiments on cube reorientation and Baoding ball swapping show that temporal tactile information can compensate for degraded visual observations (e.g., under masking), enabling more robust state estimation and better manipulation performance. Our current sensor design focuses on normal-force measurements, and our evaluation is conducted in simulation; extending to richer tactile modalities and long-horizon real-world manipulation is an important direction for future work.

A key limitation of the present hardware is that the tactile sensors measure only normal forces at each taxel. In the tasks considered in this article, rigid objects, thin-film pads, and sufficiently large normal loads cause rolling and sliding motions to manifest as clear changes in the spatial distribution and temporal evolution of normal forces across the hand. Our spatio-temporal tactile graph encoder, combined with vision and proprioception, can therefore infer object motion and contact changes from normal-force-only measurements with high reliability. However, in settings that rely heavily on incipient slip detection, low-normal-force interactions, or fine control of surface shear, direct measurements of tangential forces and torques would provide complementary information. Because the proposed dynamic tactile graph builder and fusion architecture are agnostic to the specific per-taxel feature channels, an important direction for future work is to replace the current piezoresistive taxels with multi-axis tactile elements and extend each node feature from a scalar normal force to a multi-dimensional force vector (including shear components and possibly local torque). This would allow TouchWGNN to exploit both normal and tangential tactile cues within the same spatio-temporal graph learning framework.

## Data Availability

The original contributions presented in the study are included in the article/Supplementary Material; further inquiries can be directed to the corresponding author.
